# A cancer-mimicking diagnosis of peritoneal carcinosis: report of a case of abdominal non-tuberculous mycobacterial infection

**DOI:** 10.3332/ecancer.2018.860

**Published:** 2018-08-21

**Authors:** Francesca De Piano, Filippo Savoldi, Francesca Ruju, Mariacristina Ghioni, Vanna Zanagnolo, Stefania Rizzo

**Affiliations:** 1 Postgraduate School of Radiodiagnostics, Università degli Studi di Milano, Via Festa del Perdono 7, Milan 20122, Italy; 2 Department of Radiology, IEO, European Institute of Oncology IRCCS, Via Ripamonti 435, Milan 20141, Italy; 3 Department of Pathology, IEO, European Institute of Oncology IRCCS, Via Ripamonti 435, Milan 20141, Italy; 4 Department of Gynaecologic Oncology, IEO, European Institute of Oncology IRCCS, Via Ripamonti 435, Milan 20141, Italy

**Keywords:** nontuberculous mycobacteria, abdomen, peritoneal carcinosis, computed tomography

## Abstract

Abdominal non-tuberculous mycobacterial infection is a rare condition in healthy patients. When it occurs, it leads to the appearance of typical findings of peritoneal involvement, such as thickening of the peritoneal leaflets and the omentum, ascites and enlargement of lymph nodes and of mesenteric nodules. These findings may be misdiagnosed as tumour peritoneal implants.

In case of spontaneous regression of the peritoneal involvement and ascites, as well as in the absence of malignancy, the suspicion of infectious disease, including abdominal nontuberculous mycobacterial infection, should be considered.

## Introduction

Peritoneal carcinomatosis is a condition usually associated with cancer originating from the ovary, stomach, colon, pancreas or breast. In the presence of a previous and/or simultaneous cancer, the appearance of peritoneal solid nodules usually raises the suspicion of peritoneal carcinomatosis.

However, some non-oncologic pathologies may exhibit peritoneal nodules, and may be therefore misdiagnosed as peritoneal carcinomatosis.

Non-tuberculous mycobacteria (NTM) are aerobic, non-motile and acid-fast organisms. These bacteria are widespread in the environment, especially in tap water, shower heads, soil, dust and food products, and are carried by domestic and wild animals [1, 2]. NTM infection may cause lymphadenopathy, pulmonary disease, skin infections or disseminated disease, especially in immunocompromised hosts [3]. Disseminated NTM infections can involve the lymph nodes, bone marrow, lungs, skin and the gastrointestinal tract.

Risk factors for NTM include a wide range of well-known predisposing conditions (HIV, immunosuppressive therapies, solid tumours and haematologic malignancies, presence of comorbid medical conditions such as immune-mediated inflammatory disease, and congenital disorders including specific deficiencies and genetic mutations), while other risk factors remain poorly understood.

In immunocompromised patients, typical manifestations include lymphadenopathy, fever and weight loss. In healthy patients, clinical manifestations are unremarkable and sometimes may be completely asymptomatic.

Abdominal infection by NTM is a rare manifestation of the disseminated disease and may be associated with peritonitis with a high mortality rate [4].

Nontuberculous peritonitis presents with thickening of the peritoneal leaves and the omentum, ascites, enlargement of low-attenuation lymph nodes and of mesenteric nodules [5]. These findings normally raise the suspicion of peritoneal metastases in oncologic patients; yet, in asymptomatic patients without oncologic history and with a negative oncologic workup, the finding of peritoneal nodules and ascites might raise the suspicion of abdominal infectious disease, such as from NTM.

This report describes the rare case of an asymptomatic and immunocompetent patient with abdominal NTM infection mimicking peritoneal carcinomatosis.

## Clinical case

**Figure 1.**
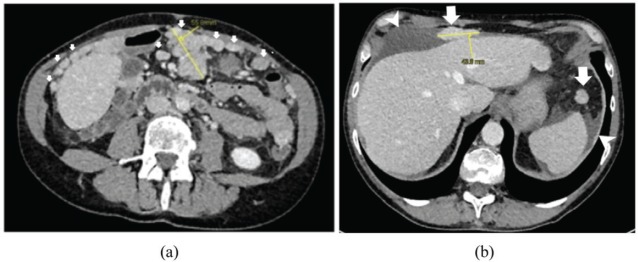
Axial contrast-enhanced CT images show multiple omental nodules [white arrows in (a) and in (b)], some of which are measured in mm [yellow callipers in (a) and in (b)] and peri-hepatic and peri-splenic ascites [arrowheads in (b)].

A 53-year-old Caucasian woman was referred to our institution because of the suspicion of peritoneal carcinomatosis, raised by the findings of ascites at a transvaginal ultrasound performed as a yearly routine exam; a pre-surgical staging exam with computed tomography (CT) scan show thickening of the gastric walls, multiple omental nodules and ascites (Figure 1).

Her previous personal history was unremarkable and she denied any clinical symptom or cancer history.

At our hospital, she underwent an esophagogastroduodenoscopy and colonoscopy, with results negative for gastric/colon cancer.

Her comprehensive metabolic profile revealed mild liver dysfunction with an alanine transaminase of 77 U/L and aspartate transaminase of 71 U/L. When tumour markers were assessed, CA125 demonstrated increased levels of 290 U/mL (normal values <35 U/mL), whereas carcinoembryonic antigen, CA 19.9 and other immunohistochemical markers were within the normal ranges. Serological assessment of HIV, hepatitis C virus and hepatitis B virus were negative.

**Figure 2.**
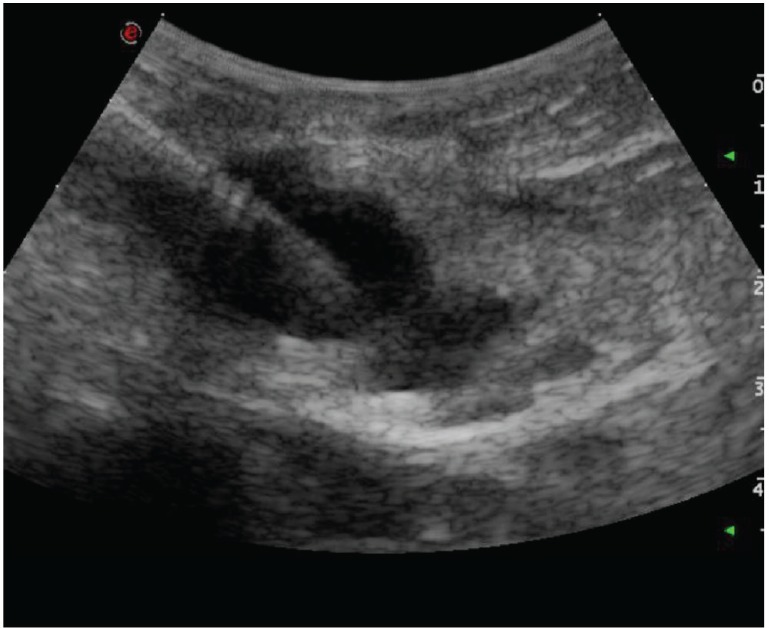
Ultrasound-guided biopsy of an omental nodule, whose diagnosis was suspicious for carcinoma with the unknown primary site.

Ten days later, the patient underwent an ultrasound-guided biopsy (Figure 2) with a diagnosis of suspicious carcinoma from the an unknown primary site.

**Figure 3.**
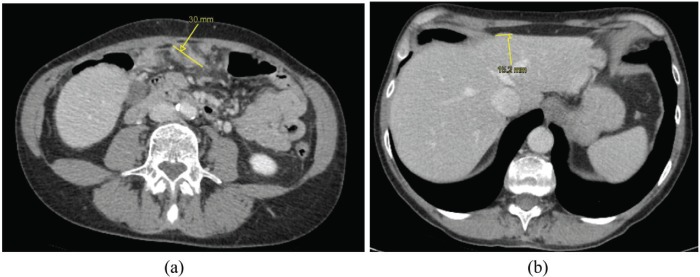
CT scan of the chest, including part of the superior abdomen, performed to complete staging after 2 weeks from the first CT scan, shows a dimensional and numerical reduction of peritoneal lesions [measures shown by yellow callipers in (a) and in (b)], as well as the resolution of perihepatic and peri-splenic ascites.

After 2 weeks, the patient received a CT scan of the thorax (to complete pre-operative staging), demonstrating a spontaneous (with no therapy) dimensional and numerical reduction of peritoneal lesions in the upper abdomen, partially included in the chest CT scan, as well as resolution of peri-hepatic and peri-splenic ascites (Figure 3).

Since there was no evidence of primary cancer at pre-operative examinations and the second CT scan revealed a partial resolution of peritoneal implants and ascites without therapy, the suspicion of an infectious disease was raised.

**Figure 4.**
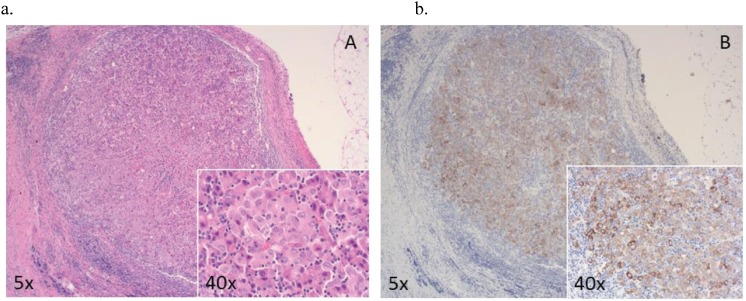
Peritoneal lymphoid aggregate with a central core of epithelioid cells (see box at higher magnification) without atypia or mitosis (a) and with only focal and weak positivity for cytokeratins AE1/AE3 (b). A lot of immunohistochemical markers were performed (WT1, S100, Ber-EP4, oestrogen receptor, p63, ALK, BRAF, CD45, CD68 and CD79) and resulted negative.

**Figure 5.**
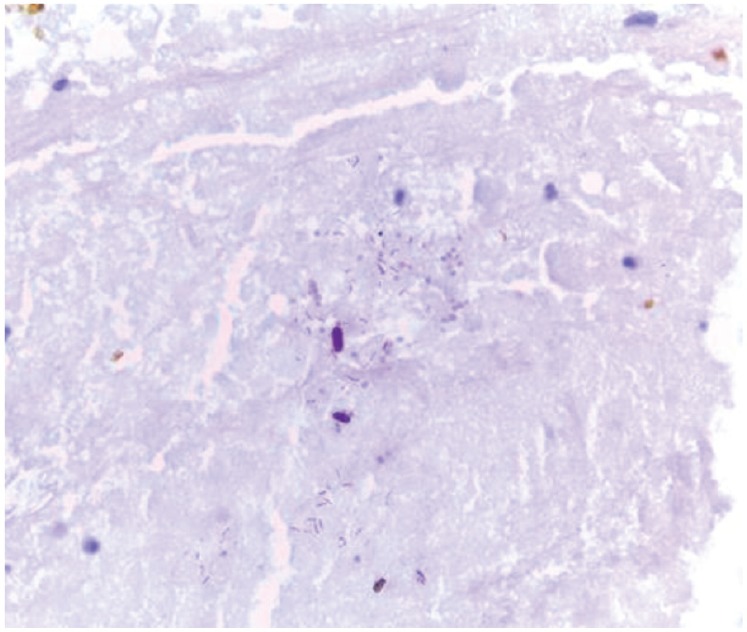
Ziehl–Neelsen stain revealed the presence of acid-fast bacilli.

The pathological evaluation of the biopsies performed on the omentum and peritoneum revealed the presence of lymphoid aggregates with a central core of epithelioid cells with large eosinophilic cytoplasm, without atypia or mitosis (Figure 4) and with no immunohistochemical marker of oncologic malignancy. Due to the presence of necrotic nodules with histiocytes and giant cells, a Ziehl–Neelsen stain was performed to identify bacilli (Figure 5), whose presence was then confirmed with molecular assays.

Because of the uncertain result of the biopsy and the conflicting results of the 2 CT scans, in order to rule out malignancy with certainty, the patient underwent a laparoscopic surgery in 2 weeks.

Definitive histological diagnosis excluded the presence of malignant cells and reported a necrotising inflammation caused by non-tuberculous mycobacteria. Hence, the patient was sent to a hospital with expertise in infectious diseases.

A follow-up CT scan performed 1 year later confirmed a complete recovery of peritoneal findings.

## Discussion

Peritoneal carcinomatosis is the intraperitoneal dissemination of any tumour that does not originate from the peritoneum itself, and it represents the most common diffuse peritoneal disease. Therefore, in cases of peritoneal solid nodules, the first diagnosis is usually peritoneal carcinomatosis. Nevertheless, there are quite a number of differential diagnoses to be kept in mind when facing peritoneal disease, such as peritoneal lymphomatosis, malignant peritoneal mesothelioma, pseudomyxoma peritonei and peritoneal infections [6].

Among peritoneal infections, NTM is an uncommon disease, but its diagnosis is mandatory because the treatment is mainly medical and should be initiated as soon as possible [3].

The recognition of patients at high risk for this disease is difficult. While some conditions are recognised risk factors, others are unknown. It is impossible to predict which patient will develop this disease because all people are exposed to NTM on a daily basis.

Frequently, this condition is misdiagnosed because the clinical symptoms and physical findings are non-specific, and the final diagnosis is based on histology.

Previous studies have demonstrated that the most common signs are fever, ascites and abdominal masses [7, 8], although these signs are unspecific.

The reported CT findings, which are not more specific after the application of post-processing optimised reconstruction algorithms [9, 10], include ascites, lymph nodes enlargement with low-attenuation, hepato-splenomegaly, diffuse small bowel thickening, liver and spleen lesions [11]. When peritonitis occurs, the CT features include diffuse thickening and multiple enlarged nodes within the mesentery. Furthermore, peritoneal nodules may change morphology over time, presenting rim enhancement in the periphery and low attenuation in the centre [11].

Based on the location and the morphology of the lesions, CT imaging can mimic many conditions, including: other infections, such as from *Mycobacterium Tuberculosis, C. Albicans* and *Aspergillus* [4]; malignancy, especially lymphoma in case of mesenteric nodal enlargement [12]; and peritoneal carcinomatosis caused by breast, gastric, pancreatic and ovarian cancer [13].

Because of the unspecific signs and symptoms, the diagnosis of abdominal NTM infection may be delayed and the lack of appropriate treatment may lead to a high rate of mortality.

The standard treatment for this disease consists of a combination of second line anti-tuberculosis drugs [14] only after a definite histological diagnosis, which usually requires invasive procedures, such as imaging-guided biopsies or minimally invasive surgery [15]. Surgical intervention is required for the management of drug-resistant infections or in cases with accumulation of pus and dead tissue; the additional medical treatment is controversial [16].

In the case reported, the first CT scan showed ascites, multiple enhancing peritoneal nodules, well depicted in greater omentum, hepatic capsule and subphrenic space, suggesting the presence of peritoneal carcinomatosis. The second CT scan showed spontaneous resolution of ascites, as well as reduction of number and size of peritoneal lesions. The changes of the lesions over time without treatment raised the suspicion of something different from malignant peritoneal carcinomatosis.

In order to rule out malignancy, a laparoscopy was performed, and the final histological diagnosis confirmed the suspicion of an infectious disease sustained by NTM.

## Conclusion

In conclusion, this report describes a rare case of an asymptomatic and immunocompetent patient with abdominal nontuberculous mycobacterial infection mimicking malignant peritoneal carcinomatosis.

Since the symptoms and CT findings are non-specific, the diagnosis may be difficult. After excluding the presence of a previous or a simultaneous tumour, an infectious aetiology should be suspected, in order to send the patient for the right treatment in a timely manner.

Hence, abdominal NTM infection should be taken into account in the presence of peritoneal nodules suspected for malignant carcinomatosis, because the treatments are different and a prompt correct diagnosis may be crucial for prognosis.

## Conflicts of interest

The authors declare that they have no conflicts of interest.

## Funding

No funding was provided to the authors for this work.
